# Environmental, social and economic perceptions of local food production: a case study of Aberdeenshire farmers’ markets

**DOI:** 10.1080/14702541.2023.2242834

**Published:** 2023-08-02

**Authors:** Jennifer Wardle, Aslam Sorathia, Pete Smith, Diana Feliciano

**Affiliations:** a School of Biological Sciences, University of Aberdeen, Aberdeen, UK; b International Business School, Teesside University, Middlesbrough, UK

**Keywords:** Farmers’ markets, local food, sustainable agriculture, perceptions, Aberdeenshire

## Abstract

Sustainable food systems are an important aspect of curbing the impacts of climate change and meeting targets of global food security. It is increasingly recognised that a wider suite of indicators is required to assess sustainability beyond the traditional environmental factors. This study focuses on Aberdeenshire, an atypical area of the UK where soils, climate and topography are not conducive to diverse or large-scale fruit and vegetable production, which in other areas, are a dominant feature of farmers’ markets. Nevertheless, Aberdeenshire needs economic diversification to offset some of the impacts of the decline in the oil and gas industry. Face-to-face questionnaires were conducted across Aberdeenshire farmers’ markets in summer 2022 to assess buyer and seller perceptions of the environmental, social and economic benefits of local food products. There was a positive attitude to local products with the majority of buyers perceiving the quality, nutrition, organic status and use of sustainable farming practices to be high. Conversely, the main products bought, baked goods and meat, are associated with negative impacts on the environment and/or human health. We discuss why, despite these shortfalls, farmers’ markets provide a valuable opportunity to distribute and promote high quality wares to support the local economy.

## 1. Introduction

The sustainability of human food systems is of increasing interest in the battle to tackle climate change. But what is a sustainable food system? For many, environmental sustainability is the predominant thought, which has often narrowly focussed on the concept of food miles (Coley et al., 2009; Stein & Santini, 2022). It is now widely recognised that food miles alone are not an adequate measure of environmental sustainability (Brunori et al., 2016; Coley et al., 2009; Smith Taillie & Jaacks, 2015; Stein & Santini, 2022). Indeed, there is evidence that some long-distance food systems are more sustainable than local due to factors including existing land use and management, pressure on local resources, and availability of sustainable transport options (Brunori et al., 2016; Saunders et al., 2009). The FAO (2018) state that to achieve sustainable food systems, collaboration is required across sectors and disciplines from local to global scales, and that by considering systems as a whole, multiple policy objectives can be achieved simultaneously.

Evolution in attitudes towards the environmental sustainability of food systems has resulted in it becoming more inclusive of biodiversity, water stress, soil health and the impacts of processing and storing. Moreover, socio-economic sustainability and governance are increasingly included in the dialogue around food systems (FAO, 2018), with Brunori et al. (2016) pointing out that sustainability is becoming more inclusive of ecological and ethical considerations and is more explicit about human health. While these further considerations are essential for assessing true impacts of the food system, they inevitably introduce higher levels of complexity and ambiguity around sustainability. For example, Schmitt et al. (2016) found local cheese production scored higher for biodiversity, soil management, animal welfare, and wealth distribution than global cheese production, but the global model fared better for efficiency, affordability, waste reduction and disposal.

Some argue that there is a divide between localists and globalists regarding sustainable food systems (Brunori et al., 2016). A key problem with this debate is the absence of definition as to what local food production actually is (Aprile et al., 2016; Pearson et al., 2011). ‘Local’ can refer to speciality foods which are promoted for their locality, but exported globally, as demonstrated by foods with Protected Geographical Indication (PGI) status (Brunori et al., 2016; Schmitt et al., 2016). This suggests that what is often referred to as ‘local food’, may be better described as ‘short food supply chain’ (SFSC).

Renting et al. (2003) divided SFSCs into three categories, (i) face-to-face – consumers have direct contact with the producer through farmers’ markets (FMs), farm shops, food boxes, etc., (ii) proximate – slightly more complex chains still focused on local or regional production and sales, often involving co-operatives, and (iii) extended – the geographical distance between producer and consumer may be great, but products are strongly linked to shared values, e.g. Fairtrade. This paper eliminates consideration of the extended category of SFSCs to focus on local products intended for local consumers at FMs.

International trade is often beneficial for global food security, as a maximum of 28% of the population could rely on key crops produced within 100 km of consumption (Kinnunen et al., 2020). It could be argued, however, that an increase in face-to-face and proximate SFSCs could increase resilience to food and nutrient insecurity in times of national or global disturbances such as war or the recent COVID pandemic (Scheelbeek et al., 2020). In these circumstances, individual movement may be restricted, and global supply chains can be severely affected through bottlenecks in maritime and terrestrial infrastructures, thus threatening national nutrient security (Macdiarmid et al., 2018).

Indeed, measurable increases in footfall and sales were reported from UK FMs during the fruit and vegetable shortage experienced in early 2023 due to climate related events in North Africa (Farm Retail Association, 2023). However, this is against a backdrop of a mass decrease in domestic production and increased reliance on imports from climate vulnerable countries, with the UK’s contribution to its domestic fruit and vegetable supplies dropping from 42 to 22% between 1987 and 2013 (Scheelbeek et al., 2020). Furthermore, the Committee on Climate Change (2019) suggests the UK should decrease its agricultural land area by 20% to accommodate afforestation projects and other carbon reducing activities. The situation highlights some of the conflicts and complexities around national food security and global considerations of sustainability.

Recently, SFSCs have thrived in many European countries, often in the form of FMs. Advantages of FMs include a fairer price for farmers, access to fresh and seasonal produce for consumers and sense of belonging (European Parliament, 2016). Scotland is disadvantaged in terms of varied local produce typical of FMs, with 85% of agricultural land categorised as ‘Less Favoured’, with the majority ‘Severely Disadvantaged’ (Scottish Government, 2021). Scotland is, however, suited to rearing livestock and maintains an unusual position in Europe whereby livestock is predominantly reared for meat production (Vosough Ahmadi et al., 2015). Scottish cattle have a good reputation, are free-from bovine tuberculosis (Scottish Government, 2021), and demand high prices on the European market (Cook et al., 2016).

Aberdeenshire is an important part of this market, with NE Scotland comprising only 16% of Scotland’s agricultural land, but accounting for 33 and 42% of cattle and sheep slaughters (Cook et al., 2016). Aberdeenshire’s largest area, covering 29.6% of land, is categorised as 3.2 in the land capability for agriculture (LCA) system. This is defined as ‘capable of average production though high yields of barley, oats and grass can be obtained’ (Macaulay Institute, 1981; Scottish Government, 2017) and is followed by 21.4% of land categorised as 3.1, suitable for high yields of a narrow range of crops. Only 2.2% of Aberdeenshire's land is capable of producing a wide range of crops. The region is therefore most suited to cereal crop production due to its soil and environmental characteristics, with the area making constructive use of its agricultural potential through being a prominent producer of malting barley for Scotland’s whisky production. This is reflected in Aberdeenshire having almost triple Scotland’s average employment rate in agriculture, fisheries and forestry at 4.38% compared to 1.66% (Cook et al., 2016).

With COVID restrictions and associated issues of food security and mental health impacts recently in mind, we sought the opinions of market attendees (consumers) around Aberdeenshire on the environmental, social and economic benefits of local food and FMs, as well as gaining initial insight from producers about their challenges regarding FMs. The study was restricted to the researchers’ home region of Aberdeenshire to add resilience, as concerns about new Coronavirus strains and associated travel restrictions were prevalent during the planning process. In this study, the term ‘local’ rather than ‘short supply chain’ was used as it was assumed to be a more user-friendly term. In the context of questioning people about the food available on the FMs they were attending, it was assumed that participants would think in terms of our intended use of the word ‘local’, i.e. face-to-face SFSCs. The wider aspects of sustainable food, were considered by asking questions explicitly relating to nutrition and other socio-economic factors such as pricing and general enjoyment of the FMs.

## 2. Methods

### 2.1. Study area

Aberdeenshire has a population of ∼261,000 and a low population density of 41 people per km^2^ (ONS, 2020). The largest age bracket is 50–59 year olds, accounting for 15.3% of the population. It is an affluent area with the 2021 median annual wage amounting to £32,605 compared to £31,659 in Scotland and £31,285 for the UK (NOMIS, 2021). There is also a higher percentage of economically active people at 80.2%, compared to 77.1% in Scotland and 78.6% in Great Britain (NOMIS, 2022). The economy is largely reliant on the energy industry, farming and fisheries. Over recent years there has been a decline in the oil industry, and the beef industry, both of which are viewed as being environmentally unsound due to high greenhouse gas emissions and other associated pollutants. There is a need, therefore, for economic diversification. Aberdeenshire traditionally has less farm-based economic diversity than neighbouring regions due to the previous availability of lucrative off-farm work in the oil industry, but is rich in high quality local produce (Cook et al., 2016).

### 2.2. Questionnaire

Sixty face-to-face questionnaires (Supplementary material) were conducted at six Aberdeenshire FMs throughout June and July 2022 (Table 1), all of which occur on one Saturday of the month. A seventh market was visited in Stonehaven but was excluded due to heavy rain deterring customer participation. Each market was visited once for the duration of the market, with questionnaires conducted with both buyers (*n *= 44) and sellers (*n *= 16). Buyers were targeted by approaching every fifth customer near a specific stall. At least two food sellers were targeted at each FM, with selection based on their availability and causing minimum disturbance to sales. Participation was affected by weather conditions and customer age, with lower response levels on rainy days and amongst younger customers.

**Table 1.** Sampled farmers’ markets and conditions on the day.

Town & venue	Size	Fruit/vegetable stalls	Stall types	Weather
Aboyne Green	Medium-large	Yes	Baked goods, meat, seafood, cheese, preserves, plants, vegetables	High wind, slightly sunny
Banchory (car park)	Medium	Yes	Meat, fish, cheese, baked goods, preserves, plants, worms, fruit	Sunny
Inverurie Town Square	Medium	No	Meat, fish, cheese, baked goods, preserves, crafts, books, homeware	Slightly sunny
Ballater Church Green	Medium	Yes	Meat, cheese, vegetables, baked goods, preserves, plants, local gin, crafts	Sunny, windy
Ellon (Neil Ross Square)	Small-medium	No	Meat, fish, cheese, baked goods, preserves, plants	Cloudy to sunny
Macduff Primary School Hall	Small-medium	Yes	Meat, cheese, vegetables, baked goods, homeware, fruit, preserves, crafts, clothing	Windy, rainy, sunny

### 2.3. Likert and statistical analysis

Five Likert items were included in the questionnaire, four of which gauged attitudes of FM goods in comparison with supermarket goods. Each Likert item had five options which were assigned numerical values from 1 to 5, with 1 relating to strongly disagree and 5 relating to strongly agree. A weighted score was established for each individual question by multiplying the frequency of each possible response by its assigned value before diving it by the number of responses. The Likert scale sentiment score was established for each individual buyer by taking the mean of their responses relating to satisfaction of the FM compared to supermarkets (Likert, 1932). Kruskal Wallis and chi-squared tests were used to establish if there were significant differences in satisfaction based on sex, age, education level and distance travelled. Analysis was conducted in Excel and R v4.2.1 with R’s simulate.p.value function used when numbers were low in some categories. This function initiates a Monte Carlo test with 2000 iterations to calculate the *p* value, reducing potential error when sample sizes are small or contain zero values.

## 3. Results

### 3.1. Farmers’ market attractions

**Figure 1.**
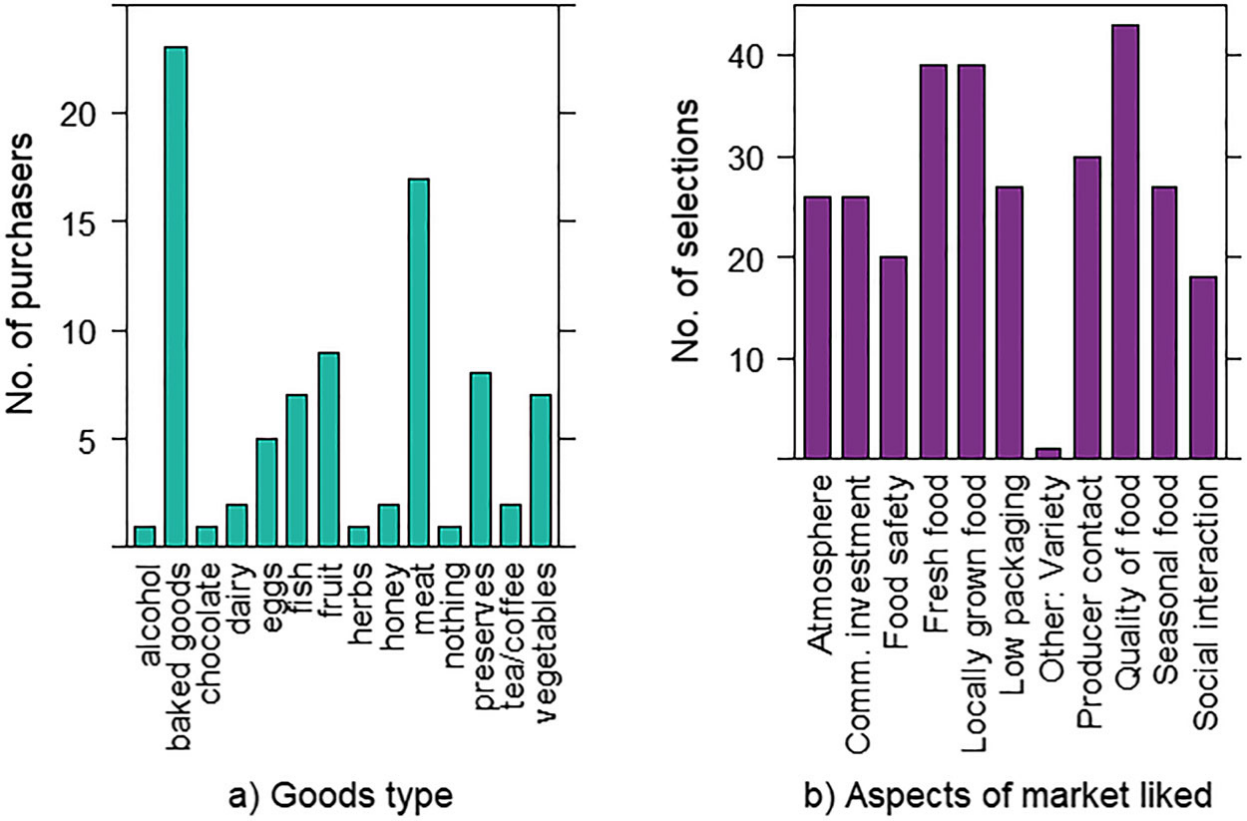
(a) Food types purchased, and (b) Aspects of farmers’ markets valued by customers.

The majority of consumers (86.7%) felt that the quality of food on the markets was higher quality than supermarkets. A lower percentage (61.4%) felt that this was reflected in a higher price, although 29.6% believed the price matched supermarkets. The quality of food was the most frequent customer answer when asked which aspects of the FMs they liked, with 95.5% selecting this option. This was followed by access to locally grown food and seasonal food, both mentioned by 86.4% of consumers (Figure 1). While direct contact with the producer ranked 4th, selected by 65.9%, social interaction was cited by only 38.6% of consumers. Only one person independently stated social interaction as a prime motivator for attendance in an open question and it was observed that many customers seemed hurried and did not spend long at the markets. When buyers explained in their own words what they liked about the market, the most common theme was support for the local community, mentioned by 56% of buyers.

The most frequently bought goods were baked goods, purchased by 52.3% of buyers, followed by meat (38.6%) and fruit (20.5%). Customers in the 50–59 age range bought a wider variety of goods than other age groups, averaging 2.3 item types (e.g. meat, baked goods etc.) if excluding the sole representative of the under 20s group who bought 4 item types. N.B. under 20s may have been underrepresented due to targeting adults, although appeared to be a minority group irrespectively.

### 3.2. Distance travelled to farmers’ markets

**Figure 2.**
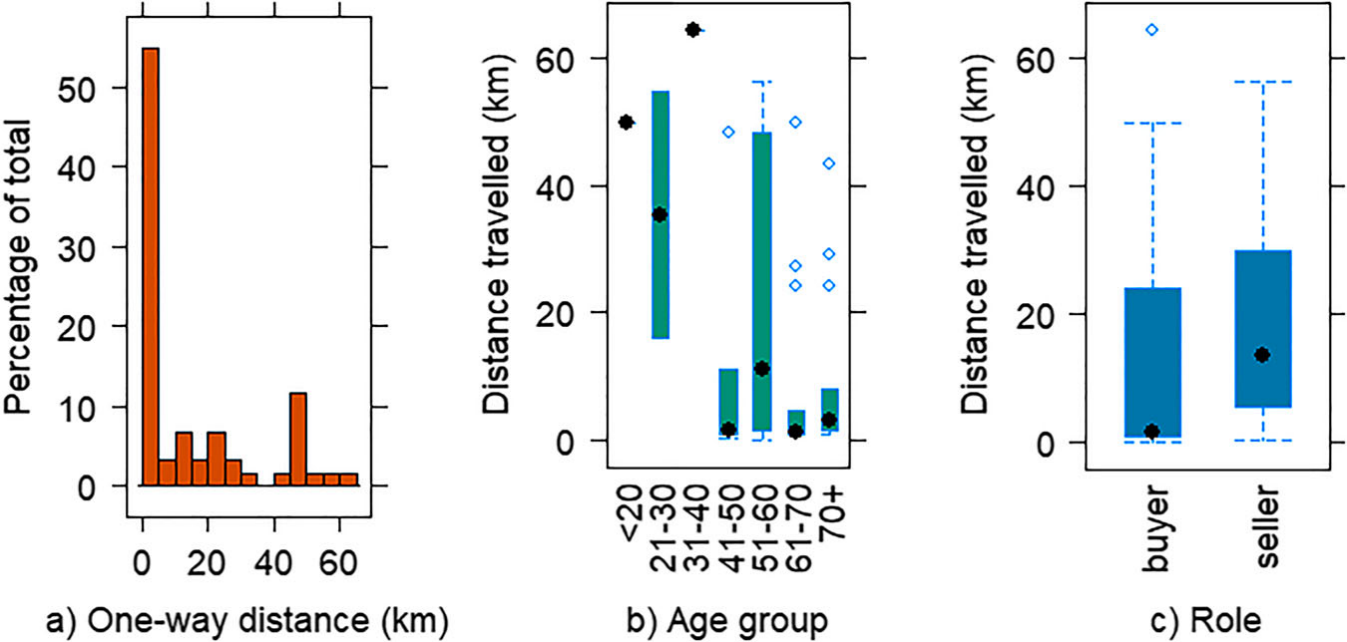
One way distance travelled by (a) frequency, (b) age group, (c) role in km. Dots indicate median value.

Over half of the respondents travelled less than 5 km one-way to the markets (Figure 2(a)). There was a significant difference (*p* = 0.03) in distance travelled by age group (Figure 2(b)), with younger people travelling further than older people, with a marked difference between under 40s and over 40s. Sellers generally travelled further than buyers, with respective one-way means of 20.28 (SD 18.74) km and 13.25 (SD 19.42) km. As indicated by the large standard deviations, there was a wide range within groups, with median values being considerably less at 13.70 and 1.6 km respectively (Figure 2(c)). The difference in distance travelled between the six FMs was noticeable though not statistically significant, with means between 11 and 11.5 km for Banchory, Ballater and Ellon, and at 24.55 (SD 23.80) km for Aboyne.

### 3.3. Likert items and Likert scale

**Figure 3.**
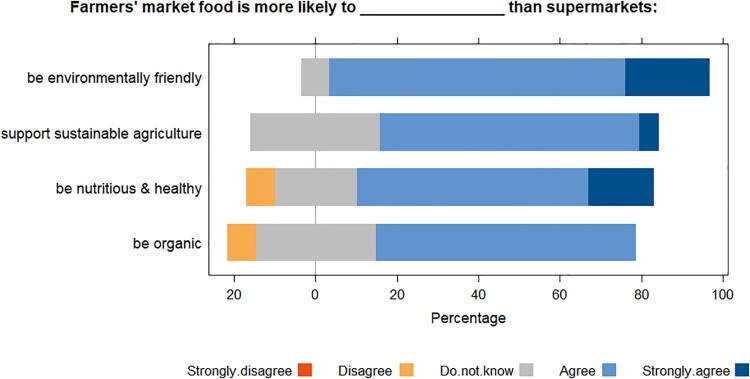
Sentiments of health and sustainability indicators of farmers’ market goods. ‘Strongly disagree’ was an option but not selected by anyone.

Attitudes of buyers towards FMs goods were positive, with Likert weighted mean scores all rounding to 4 (Table 2), the assigned code for ‘agree’. Respondents perceived that FM food was more nutritious and healthier, more environmentally friendly, more likely to be organic and produced through more sustainable agriculture than supermarket food (Figure 3). The most positive perceptions related to environmental sustainability which had a weighted mean score of 4.14. Nobody strongly disagreed with the statements about FMs for any of the Likert-item questions. The overall Likert scale score from the mean of the four questions was 3.81 (SD 0.24) of a possible 5, indicating agreement with positive statements about the market. The importance of where fruit and vegetables are grown ranked closer to neutrality with a weighted mean of 3.41. This is in fitting with the importance of who produced the food, with the majority answer being that it was of low importance (45.5%).

**Table 2.** Likert weighted mean for each item.

Question	Nutritious	Environmentally friendly	Organic	Sustainable agriculture
Weighted mean	3.82	4.14	3.57	3.73

In addition to calculating Likert scores for each question, mean scores of the four similar Likert items, namely nutritious and healthy, environmentally friendly, organic and support sustainable agriculture, were calculated for individual respondents. There was no significant difference in people’s perceptions towards the market produce when grouped according to sex (*p* = 0.89), education level (*p* = 0.65), number of people in household (*p* = 0.08) or distance travelled to market (*p* = 0.50) at a significance level of 0.05. There was, however, a significant difference according to age (*p* = 0.04), with the 41–50 age group scoring highest at 4.31, compared to the lowest mean value of 3.63 for the 61–70 age group.

### 3.4. Frequency of local and organic purchases

The proportion of respondents who frequently bought local food was considerably higher than those who frequently bought organic food at 51.16% and 16.28% respectively. However, this pattern is reversed when looking at those who purchased them ‘sometimes’ at 41.68% and 62.79%. Only 2.33% never bought local, whereas 18.60% never bought organic. There was no significant difference in purchase frequency of either organic or local produce between age groups, sex, education level or distance travelled with the exception of distance travelled and frequency of local purchases (*p* = 0.05). Those who claimed to ‘always’, ‘frequently’, ‘sometimes’ or ‘never’ buy local had corresponding distances of 2.0, 13.3, 12.4 and 49.9 km respectively, although only one person was in the latter category.

### 3.5. Seller perspectives

Seller-focused aspects of the questionnaire were limited to two questions beyond basic demographics as they were busy working. Fourteen out of sixteen sellers had been to the markets several times. When asked about difficulties they encountered as sellers on the market, the most popular answers were that there were no difficulties (7), followed by the weather (6) and maintaining food shelf-life (6). Only one seller sold exclusively at FMs, with the most common venues for sellers being their own shop, hotels, fast-food restaurants and grocery stores, all of which had four responses each.

## 4. Discussion

### 4.1. Distance to food supplier

All except two sellers travelled from within the 30-mile radius of production specified by UK Parliament (2009) for the Certified Farmers’ Market scheme, with half travelling 7 miles/11.3 km or less. Customer distances were wider ranging between 0.05 and 64 km (0.03–40 miles). Coley et al. (2009) suggest that when consumers drive over 6.7 km round-trip to a small farm shop, carbon emissions are greater than those from large-scale vegetable box deliveries which entail cold storage, packing, and transport to regional hubs and customer homes. Home-deliveries therefore potentially offer reduced environmental impacts for approximately 40% of Aberdeenshire FM buyers. National schemes, however, omit the attractions of locality and pre-purchase checks of quality, and are potentially subject to contentious Highlands and Islands premium delivery charges affecting rural Aberdeenshire. In addition to frustrating residents, the prices suggest longer supply-chains are less efficient in this area, potentially due to being inaccessible to HGVs. In this sparsely populated area, weekend market days in main towns where a range of other shops and services could be visited on the same trip, may be the most practical option. Other studies have found that weekend FMs are considerably more successful than weekday FMs, attracting more sales and drawing in clientele from a greater distance (Garner & Ayala, 2018; O’Hara et al., 2022).

### 4.2. Perceptions of farmers’ market goods

Findings that customers perceived the food products to be high quality, nutritious, organic and produced by sustainable agricultural practices are typical of studies of farmers’ markets and other SFSCs (Aprile et al., 2016; Gumirakiza et al., 2014; Pearson et al., 2011). This is perhaps unsurprising given that sampling was done amongst FM clientele. Quality is an ambiguous term, with commercial quality relating to appearance, and nutritional quality focusing on nutrients and other bioactive compounds (Edwards-Jones et al., 2008). These qualities degrade from the point of harvest or slaughter through cellular respiration or oxidation, exacerbated by damage from rough treatment (Bouzari et al., 2015; Domínguez et al., 2019; Rickman et al., 2007). Food products for local FMs are often harvested at peak maturity, specifically for the market, making them fresher than those for longer supply chains (Koszewski et al., 2010). Given these considerations, it is reasonable to surmise that the SFSC and direct contact with the producer who is accountable, result in the produce being fresher, more nutrient-rich and less damaged than those from more distant, longer supply chains.

Assessments of perceptions against reality are, however, scarce, with no literature found to compare the nutritional composition of FM fresh goods compared to supermarkets. It is therefore unknown whether these perceptions are founded, although based on visual attractiveness alone, Millichamp and Gallegos (2013) found no significant difference between fruit and vegetable products from FMs and supermarkets. Gumirakiza et al. (2014) suggest that more enforcement of policies around food standards at FMs would increase customer confidence and be beneficial for trade. Literature on the sustainability of agricultural methods for FM produce is equally scarce, although Schoolman (2019) found that fewer chemical inputs were used for produce for local consumption than conventional farming, but differences were shrinking over time.

Consistent with literature on FMs (Farmer et al., 2017) and local food (Aprile et al., 2016; Brunori et al., 2016), prices were perceived to be high. Social justice and unequal access to high quality SFSC food are often cited as concerns with FMs, with clientele often deemed to be educated, affluent and white (Garner & Ayala, 2018; Smith Taillie & Jaacks, 2015). While prices are assumedly restrictive for some, Aberdeenshire largely fits the stereotype demographic of where FMs are likely to thrive; it is affluent, educated and one of the UKs most ethnically homogenous areas, with 98.5% of the population identifying as white (Scottish Government, 2011).

Notwithstanding the shortcomings in accessibility, the demographic that most favoured the FMs in our study, i.e. 50–59 year olds in an affluent area, arguably have the greatest capacity for positive environmental impact by shopping locally. This is due to this demographic being the most likely to purchase fruit and vegetables from climate vulnerable countries, including those with a high-risk of water scarcity (Scheelbeek et al., 2020). However, for fruit and vegetable consumption changes to occur in Aberdeenshire, there needs to be more availability of these goods on the markets. Garner and Ayala (2018) found that these fresh goods were what FM customers were requesting more of.

#### 4.2.1. Perceptions of health and environmental benefits

There are arguments that FMs generally encourage the consumption of fresh fruit and vegetables. Smith Taillie and Jaacks (2015) claim that reliable evidence of this is scarce, but Kelley et al. (2022) used skin biomarkers to objectively test self-reported claims from FM attendees that they ate more fruit and vegetables as a result of the markets, finding their claims to be true. Despite the consensus in Aberdeenshire that FM food was nutritious and healthy (Figure 3), some markets did not sell fruit or vegetables, but all had multiple stalls for meat-dominated animal products and baked goods, which were the most frequently purchased products.

This has negative implications for human health since consumption of fruit and vegetables is well below recommended levels, with only ∼30% of adults in the UK eating 5-a-day (Scheelbeek et al., 2020). Conversely, there is overconsumption of free sugars, which are common in baked goods and associated with weight gain, diabetes, cardiovascular disease and some cancers (FSS, 2018; Tilman & Clark, 2014). No age-group in the UK currently meets the recommendation of obtaining less than 5% of energy from free sugars (PHE & FSA, 2018).

The negative impacts of red meat on human health are well known, though limited quantities can be beneficial for providing nutrients such as iron and calcium (FSS, 2018). The high environmental costs of ruminant livestock arise from several factors including greenhouse gas emissions, eutrophication and land acidification (Clark et al., 2022; Poore & Nemecek, 2018; Ripple et al., 2014). When assessing environmental impacts based on a composite score of four indicators with a maximum of 100 for the worst-case scenario, Clark et al. (2022) found the greatest impacts from beef and lamb at 30, with that from fruit and vegetables being less than 2, with the latter also being considerably more nutritious. Given the UK’s already high level of meat consumption and diet-associated chronic disease (Macdiarmid et al., 2018), Aberdeenshire FMs do not appear to meet sustainability criteria in terms of human health.

Motivations for visiting FMs are therefore not entirely straightforward, with even the term ‘farmers’ market’ appearing to be a misnomer to some extent, due to the heavy presence of baked goods and other non-farmed items. The products were perceived as environmentally friendly and nutritious, but popular purchases arguably did not meet those criteria. Also, while Farmer et al. (2017) found that FMs offer social interaction and leisure activity, the majority of participants expressed no interest in this and customers generally appeared to be hurried. This may be a result of shopping at FMs becoming a normal experience rather than a novelty, which would essentially be more beneficial for producers. It appears that perceived high quality and supporting the local community prevailed over other influences, though more research is needed on the appreciation of small-batch products, how FMs fit into wider consumption patterns, demand for healthier local products, and the extent farmed products conform to consumer perceptions.

### 4.3. Capacity for increasing fruit and vegetable production

While culture and lifestyle choices are important considerations, increasing the prominence of fresh local vegetables may help ameliorate their consumption deficiency. As well as these healthy foods being absent from some FMs, there is a deficiency of fruit and vegetable imports to meet nutritional guidelines (Macdiarmid et al., 2018). Given Aberdeenshire’s limited scope to commercially grow more than a narrow range of crops (Macaulay Institute, 1981; Scottish Government, 2017), it is unsurprising that already-stretched farmers do not diversify vegetable production, which may prove unprofitable. Market demand has been perceived as a limiting factor for experimental new practices due to the associated risks and the smaller margins faced by farmers, that do not allow failure (Feliciano, 2022). As demonstrated by the growth in allotments and community gardens, there is, however, potential to grow a reasonably wide range of fresh goods on a smaller scale. Supported by policy interventions, this could provide a niche for market gardeners, or open opportunities for community schemes which pool excess allotment produce to sell on a not-for-profit basis. If coupled with FM coupon systems for low income households which have proved effective in other countries (Caron-Roy et al., 2022), this could expand the customer base, increasing sustainability in economic terms as well as social dimensions relating to human health and ethics.

### 4.4. Aberdeenshire’s local context

With meat, particularly beef, being of major importance to Aberdeenshire’s economy, it is logical to promote meat products and gain the premium prices available on local FMs to increase the resilience of the area’s cultural background. As well as the PGI status of ‘Scotch lamb’ and ‘Scotch beef’ ensuring traditional grass-feeding systems are maintained (Scottish Government, 2021), it assures short distances travelled for livestock travel between birth and slaughter, therefore having positive impacts on animal welfare (Brunori et al., 2016). Coupled with the extensification of cattle-farming in the area (Cook et al., 2016), social and ethical sustainability are arguably strong, with environmental sustainability potentially above standard for ruminant meat production due to grazing systems having a lower water footprint than industrial systems (Smith Taillie & Jaacks, 2015). It can thus be argued that Aberdeenshire farmers are making the most of the specialist opportunities available.

Additional benefits that could be gained from local branding and high-quality products being promoted and made regularly available is the potential for it to increase tourism (Pearson et al., 2011) and agrileisure (Farmer et al., 2017) which bring their own economic advantages. In conjunction with NE Scotland playing a dominant role in supplying barley for whisky production (Cook et al., 2016), with a number of distilleries, and the attractions of seaside towns and the Cairngorms National Park, Aberdeenshire is well-positioned to become a more prominent tourist destination. Farmers’ markets can add value to this potential by facilitating and promoting high-quality local products. Indirectly, by helping to secure farmer livelihoods, traditional, scenic rural landscapes are maintained.

### 4.5. Wider considerations on the sustainability of food systems

The overall sustainability of local food systems is highly variable and dependent on the metrics used. Even specific environmental measures such as carbon footprints are difficult to quantify (Clark et al., 2022). Ironically, the potential to lower the carbon footprint of food systems by SFSCs may conflict with the UK’s 2050 net zero targets as the production of imported food is not accounted for in the UK GHG emissions inventory, whereas locally produced food inevitably is. Production-based emission accounting rather than consumption-based emission accounting essentially lets the UK government and consumers off the hook by transferring the negative externalities to the producing nation. Therefore, even when the true carbon footprint of food systems may be reduced by SFSCs, UK greenhouse gas emissions would be increased, potentially disincentivising policy makers to act for the greater good.

Aprile et al. (2016) discuss how policy makers, food producers and the EC were evaluating the potential of adopting policy tools to assist with marketing products on a regional level and making the added value of local food more explicit. This would likely be a welcome intervention given the inference that farmers are skilled in growing food but not in marketing to suit local consumers or customer interaction (Garner & Ayala, 2018). With most FM studies, including this one, focusing on consumer perceptions, more detailed research is required regarding farmer challenges with FMs, other SFSCs, and the importance of them to farmer incomes.

## 5. Summary

Farmers’ markets hold benefits for local economies, create opportunities for social interaction and have the potential to improve diets. In this study, direct social interaction was not highly valued, although socio-economic support for local businesses was a strong motivator for attendance. The Aberdeenshire FMs were highly valued for the quality, freshness and locality of goods, and as a means of strengthening local communities. Conversely, outcomes for health and the environment appear less favourable. As baked goods and meat were the predominant purchases, benefits to human health through increased consumption of fruit and vegetables were not apparent. To some extent this can be attributed to environmental conditions not being conducive to growing a broad range of fresh goods on a large scale, with production potentially demanding more creativity around collectives of small-scale production. Nevertheless, under-consumption of fruit and vegetables, and over consumption of free sugar and red meat are key concerns of the Scottish diet and the area would benefit from policy drives to make fresh fruit and vegetables more available and accessible to those on low budgets. Although farming of ruminant animals has negative consequences for the environment and human health, the standards of animal welfare, environmental conditions and quality of meat associated with Scotch PGI status are relatively high. Aberdeenshire FMs facilitate farmers to benefit from the region’s unique circumstances and are supported by locals who place importance on high-quality local products.

## Supplementary Material

Supplemental Material

## Data Availability

Data available from Mendeley Data doi:10.17632/f5tk867d65.1.
