# Supplementary material for: Environmental, social and economic perceptions of local food production: a case study of Aberdeenshire farmers’ markets
Source: Scott Geogr J. Author manuscript; Available in PMC 2024 May 21. (PMC7615968; doi:10.1080/14702541.2023.2242834)
Supplement: Supplementary Material [file EMS188509-supplement-Supplementary_Material.docx]

**Supplementary material for Environmental, social and economic perceptions of local food production: A case study of Aberdeenshire farmers’ markets**

MARKET NAME OR LOCATION: ____________________

1. **Age group**

- Under 20
- 21-30
- 31-40
- 41-50
- 51-60
- 61-70
- Over 70

1. **Gender**

- Male
- Female

1. **Education**

- Primary School
- Secondary education
- Further education
- Higher education
- Other ___________________________

1. **You are**

- Food seller
- Food buyer

Other_________________________________

1. **What is the number of people living in your household?** ________________
2. **How often do you make visits to local market?**

- I am a first-time visitor
- I have been few times
- I have been several times

1. **How long have you been visiting this market? (in years, months, or weeks) ________________**
2. **How far (in miles) have you travelled to this market? _____________________**
3. **What do you like about local farmer’s market? Multiple options possible**

- Atmosphere of the market
- Direct contact with the producer
- Access to seasonal food products
- Social interaction
- Lower price
- It is an investment in our community
- Access to locally grown food
- Availability of fresh food
- Quality of the food
- Food safety
- Less packaging involved than supermarkets
- Other________________________

1. **How much do you agree with the following sentences?**

- Food from local markets is more nutritious and healthier that food from supermarkets

1 = strongly disagree

2 =disagree

3 = unsure

4 = agree

5 = strongly agree

- Food sold at local markets is more environmentally friendly

1 = strongly disagree

2 =disagree

3 = unsure

4 = agree

5 = strongly agree

- Food sold at local markets is more likely to be organic

1 = strongly disagree

2 =disagree

3 = unsure

4= agree

5= strongly agree

- Food at local markets supports sustainable agricultural practices

1 = strongly disagree

2 =disagree

3 = unsure

4= agree

5= strongly agree

1. **What kind of foods will you buy from a local market? Multiple options possible**

- **S**easonal fruits
- Seasonal vegetables
- Homemade baked goods
- Homemade jams and preserves
- Local honey
- Farm-fresh eggs
- Dairy (milk, cheese)
- Local meats
- Fish
- Game
- Other_________________________________________________________

1. **How much do you agree with the sentence: When purchasing fresh fruits or vegetables I do not care where they are grown:**

- Strongly agree
- Agree
- No opinion
- Disagree
- Strongly disagree

1. **How does food quality at local markets compare to food quality at supermarkets?**

- Quality is higher
- Quality is the same
- Quality is lower
- Do not know

1. **How do prices at local farmers' market compare to supermarket?**

- Price is higher
- Price is the same
- Price is lower
- Do not know

1. **How often do you purchase local food?**

- Always
- Frequently
- Sometimes
- Never
- Do not know what local food is

1. **How often do you purchase food which is labelled `organic'?**

- Always
- Frequently
- Sometimes
- Never
- Do not understand meaning of `organic'

1. **When purchasing food, how important is to know who produces it?**

- Not important
- Fairly important
- Important
- Very important
- Extremely important

1. **What foods have you bought from this local market?**

**_____________________________________________________________________**

**_____________________________________________________________________**

1. **Would you like to write/say in your own words why do you buy food in this local market?**

**_____________________________________________________________________**

**_____________________________________________________________________**

1. **(For sellers) which are the main difficulties from selling at local markets? Multiple options possible**

- Understanding rules and regulations
- Supply window
- Maintaining shelf life
- Low customer interest
- Weather and other factors
- Negotiate a purchase price
- Purchasing enough for shelf space
- Produce arrives ready for sale
- Meeting local growers
- None
- Other_____________________________________

1. **(For sellers) are you selling to other than local markets? __________**

- Grocery stores
- Hotels
- Fast food restaurants
- Schools and colleges
- Others____________________________
